# Mesenchymal Stem Cells Modified with a Single-Chain Antibody against EGFRvIII Successfully Inhibit the Growth of Human Xenograft Malignant Glioma

**DOI:** 10.1371/journal.pone.0009750

**Published:** 2010-03-18

**Authors:** Irina V. Balyasnikova, Sherise D. Ferguson, Sadhak Sengupta, Yu Han, Maciej S. Lesniak

**Affiliations:** The Brain Tumor Center, The University of Chicago, Chicago, Illinois, United States of America; City of Hope Medical Center and Beckman Research Institute, United States of America

## Abstract

**Background:**

Glioblastoma multiforme is the most lethal brain tumor with limited therapeutic options. Antigens expressed on the surface of malignant cells are potential targets for antibody-mediated gene/drug delivery.

**Principal Findings:**

In this study, we investigated the ability of genetically modified human mesenchymal stem cells (hMSCs) expressing a single-chain antibody (scFv) on their surface against a tumor specific antigen, EGFRvIII, to enhance the therapy of EGFRvIII expressing glioma cells *in vivo*. The growth of U87-EGFRvIII was specifically delayed in co-culture with hMSC-scFvEGFRvIII. A significant down-regulation was observed in the expression of pAkt in EGFRvIII expressing glioma cells upon culture with hMSC-scFvEGFRvIII vs. controls as well as in EGFRvIII expressing glioma cells from brain tumors co-injected with hMSC-scFvEGFRvIII *in vivo*. hMSC expressing scFvEGFRvIII also demonstrated several fold enhanced retention in EGFRvIII expressing flank and intracranial glioma xenografts vs. control hMSCs. The growth of U87-EGFRvIII flank xenografts was inhibited by 50% in the presence of hMSC-scFvEGFRvIII (p<0.05). Moreover, animals co-injected with U87-EGFRvIII and hMSC-scFvEGFRvIII intracranially showed significantly improved survival compared to animals injected with U87-EGFRvIII glioma cells alone or with control hMSCs. This survival was further improved when the same animals received an additional dosage of hMSC-scFvEGFRvIII two weeks after initial tumor implantation. Of note, EGFRvIII expressing brain tumors co-injected with hMSCs had a lower density of CD31 expressing blood vessels in comparison with control tumors, suggesting a possible role in tumor angiogenesis.

**Conclusions/Significance:**

The results presented in this study illustrate that genetically modified MSCs may function as a novel therapeutic vehicle for malignant brain tumors.

## Introduction

Malignant gliomas remain a challenge in neuro-oncology. Complete surgical resection is virtually impossible due their diffusely infiltrative nature and systemic therapy is limited due to the presence of the blood brain barrier (BBB). These obstacles have prompted the generation of innovative strategies to tackle high-grade brain tumors. Among such new strategies is the development of therapies that target specific cell populations and increase therapeutic efficacy without harming normal surrounding cells. Tumor specific antigens which are uniquely expressed by glioma cells but not by normal brain are excellent candidates for targeted therapies.

EGFR over-expression is one of the most common genetic alteration in primary glioblastoma multiforme (GBM) with a frequency of approximately 40% [1]–[4]. Along with amplification of the EGFR gene, there are also mutations. The most common mutation is the EGFRvIII, which is not seen in normal tissue but observed in 20–30% of patients with GBM [4]–[8]. Moreover, in patients where EGFR gene amplification is confirmed, the proportion of EGFRvIII increases up to 60% [9]–[11]. EGFRvIII is a truncated form of EGFR that does not bind a ligand and is constitutively active. It is established that the presence of this variant enhances the aggressiveness of gliomas via several mechanisms [4], [12]–[18].

The frequent expression of EGFRvIII in human cancers but not in normal tissues makes it a promising target for therapeutic applications. Examples of such therapies include tyrosine kinase inhibitors and monoclonal antibodies [4]. There have been several monoclonal antibodies (mAb) developed to target EGFR with cross reactivity to EGFRvIII. Patel et al., showed that a mAb, cetuximab (c225), successfully targets and binds to U87MG cells expressing EGFRvIII. This binding led to cetuximab-EGFRvIII complex internalization and a subsequent reduction in phosphorylated EGFRvIII in transfected cells. Also, the authors found that treatment of U87-EGFRvIII cells with cetuximab resulted in a 40–50% reduction in cell proliferation [19]. Clinical trials have been less promising. Recently, Neyns et al., conducted a phase II study using intravenous cetuximab to treat patients with recurrent high grade glioma. Cetuximab was found to have limited effect on disease progression and survival time [20]. EGFRvIII specific monoclonal antibodies have also been developed. Sampson and co-authors generated Y10, an antibody specific for EGFRvIII. They showed that Y10 was effective against EGFRvIII-expressing B16 melanoma in the brain. Specifically, intratumoral injection improved survival by 286% [21]. Mishima et al., tested efficacy of another anti-EGFRvIII antibody, mAb 806, in mice with intracranial xenografts. These authors found that after systemic treatment with mAb 806, mice with EGFRvIII expressing tumors (e.g. U87-EGFRvIII) had a 61.5% increase in median survival compared to controls [22]. Despite these encouraging results, EGFRvIII tumors eventually grew and remissions proved to be transient. The authors suspected this may have been due to inefficient distribution of antibody in the tumor bed. Additionally, these studies required large quantities of antibodies to achieve the therapeutic effect *in vivo*. Poor penetration of large antibody molecules through the BBB could also account for their low or transient therapeutic effects. A few studies have been successful in designing single-chain antibody fragments (scFv) with specific high affinity binding for EGFRvIII [23], [24]. For example, Schmidt et al., demonstrated that genetic fusion of scFv 14E1 with truncated form of Pseudomonas exotoxin A drastically reduced metastases of Renca-EGFRvIII cells and number of Renca-EGFR pulmonary tumor nodules in mice [25]. Thus, a decrease in the size of therapeutic antibodies along with the development of a new approach to deliver them to poorly accessible CNS tumors may help to overcome the existing difficulties.

In recent years, mesenchymal stem cells (MSC) have been under intense investigation as vehicles for targeted therapies to solid tumors. MSCs are relatively easy to isolate and expand *in vitro*. They possess natural tropism for the tumor and are able to migrate toward the tumor after systemic, intra-arterial or distant from tumor sites injections [26], [27]. Genetic engineering of MSCs with therapeutic proteins has been shown to prolong the survival of animals [27]–[31]. Recently, it has been shown that both MSCs [32] and neural stem cells [33] can be modified to secrete therapeutic antibodies, which can successfully target xenograft tumors. MSCs were found to survive better in tumor environment in comparison with normal brain tissue. Despite this, it has been shown that the number of inoculated MSCs in the tumor declines over the time [31]. It has also been shown that the therapeutic effect depends on the proportion of MSCs in the tumor [34], and it was suggested that several injections of MSCs might be required to achieve a prolonged therapeutic effect [35]. Therefore, the approach to increase the retention or homing of MSC in the tumors is warranted. Moreover, the modification of MSCs with antibodies might become a new strategy to deliver these therapeutic molecules which poorly penetrate the BBB. The combination of antibody-directed targeting to a specific cell population and the natural tropism of MSCs to tumor holds the promise in that respect. Recently, we demonstrated the feasibility of genetic modification of hMSCs to express scFv EGFRvIII (hMSC-scFvEGFRvIII) on the cell surface in order to enhance their targeting to EGFRvIII expressing tumors [36]. In this study we investigated whether hMSC-scFvEGFRvIII possess the enhanced properties to achieve enhanced retention in EGFRvIII expressing glial tumor. Most importantly, we evaluated the therapeutic response, in particular the growth of U87-EGFRvIII glioma cell *in vitro* and *in vivo*, upon exposure to hMSC-scFvEGFRvIII.

## Results

### Effect of MSCs expressing scFvEGFRvIII on the growth of U87-EGFRvIII cells *in vitro*

**Figure 1:**
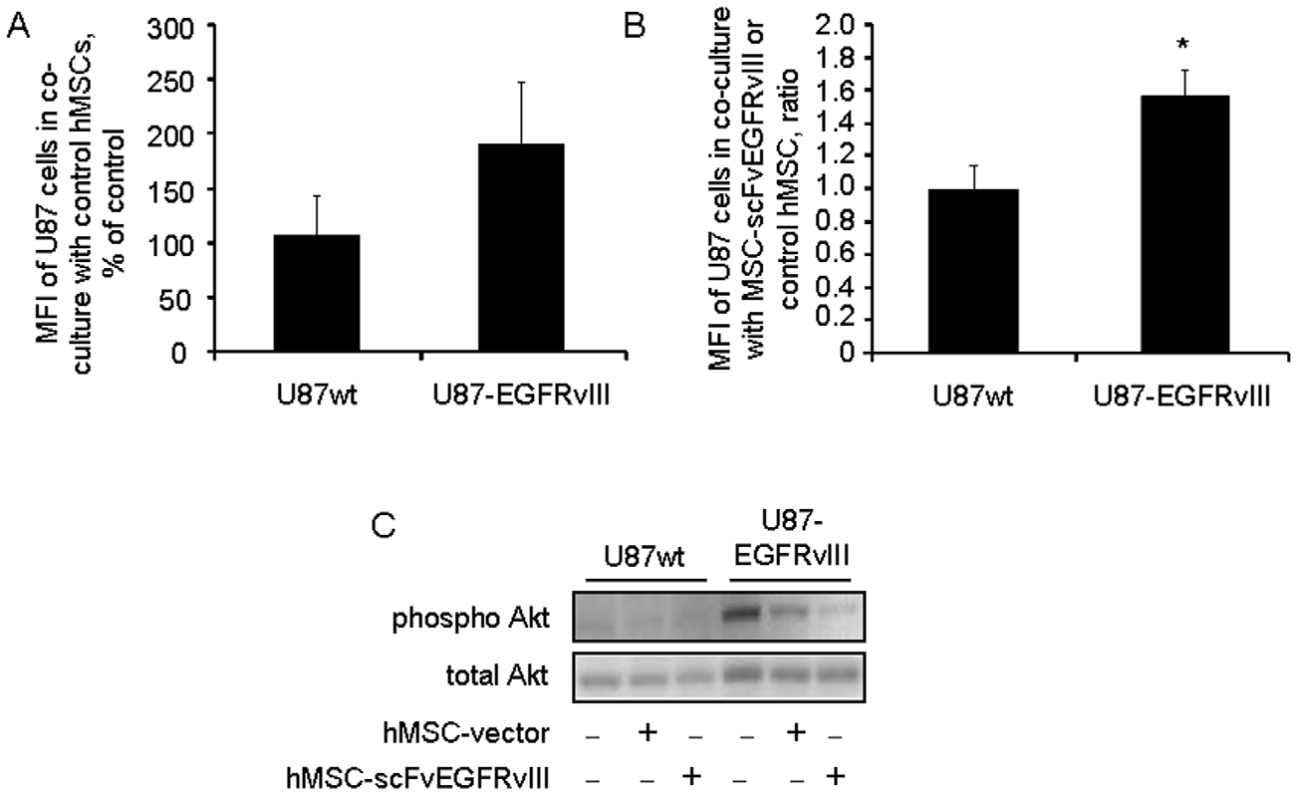
Modified hMSC-scFvEGFRvIII specifically delay the growth of U87-EGFRvIII glioma cells *in vitro* and down-regulate the pAkt expression. A- CFDA SE labeled U87wt or U87-EGFRvIII GFP negative glioma cells were co-cultured with control hMSCs for 5 days. The change in the U87 cells fluorescence was evaluated by the flow cytometry and is presented as MFI, % of control (U87 cells cultured without hMSCs). Data of three independent experiments are summarized and presented as mean ± SD. B- CFDA SE labeled U87wt or U87-EGFRvIII GFP negative glioma cells were co-cultured with control and scFvEGFRvIII modified hMSC. The changes in the U87 cell fluorescence (e.g. growth) were tracked by flow cytometry and expressed as MFI. The ratio of MFI of glioma cells co-cultured with hMSC-scFv to that with control hMSCs confirms the specific response. Data of three independent experiments are shown and presented as mean ± SD, * p<0.01. C- U87 cells expressing GFP and hMSC were co-cultured at equal ratio for 48 hours. The U87wt and U87-EGFRvIII cells were sorted out based on their GFP expression and cell pellets were processed for gel electrophoresis and Western Blot analysis. The pAkt and total Akt in cell lysates were detected using primary antibodies and developed with secondary antibodies conjugated with HRP. Representative blot of two independent experiments is shown.

We evaluated the effect of hMSC on the growth of U87 cells in co-culture experiments. hMSCs and hMSC-scFvEGFRvIII (i.e. MSCs expressing scFv EGFRvIII on the cell surface) were added at different ratios to CFDA SE labeled U87wt or U87EGFRvIII glioma cells and analyzed five days later. We observed no changes in proliferation of U87wt cells in co-culture with control or scFvEGFRvIII modified hMSC. The ratio of mean fluorescent intensity (MFI) of U87wt cells co-cultured with hMSC-scFv to that with control hMSCs was equal to 0.98 in three independent experiments. In contrast, U87-EGFRvIII cells responded by delay of growth in co-culture with control hMSCs (190±58% versus 106±36% for U87wt glioma cells). This effect was seen only at 10∶1 ratio of hMSCs to U87 glioma cells, however, the difference did not reach statistical significance (Figure 1A). Nevertheless, the delay of growth of U87-EGFRvIII cells was further enhanced in co-culture with scFvEGFRvIII modified hMSCs (Figure 1B). The ratio of MFI of U87-EGFRvIII cells co-cultured with hMSC-scFvEGFRvIII to the MFI of the cells co-cultured with control hMSCs was 1.6, suggesting that this delay in the growth of U87-EGFRvIII cells was due to specific interaction of scFvEGFRvIII with U87-EGFRvIII. In a separate set of experiments, we also evaluated if modification of hMSCs with scFvEGFRvIII influenced their proliferative potential. There was no statistical difference in the percentage of the BrdU positive cells between control and hMSC-scFvEGFRvIII cells pulsed with BrdU (4.8±0.95 vs. 5.3±0.4, respectively, p = 0.4), thus indicating a similar rate of proliferation of scFvEGFRvIII modified and control hMSCs *in vitro* (Fig. S1).

Because we observed a delay in the growth of U87-EGFRvIII glioma cells co-cultured with hMSC-scFvEGFRvIII, we hypothesized that this effect might be mediated through changes in the activity of downstream signaling molecules, in particular, pAkt. Recently, the PI3K pathway was deemed as a dominant pathway in glioma cells expressing high levels of EGFRvIII [37]. In our experiments, GFP positive U87-EGFRvIII glioma cells were co-cultured with control hMSCs or hMSC-scFvEGFRvIII for 48 hours. Glioma cells were sorted from the cell mixture based on GFP expression and subjected to gel electrophoresis and Western Blot analysis. Figure 1C shows the decrease in pAkt in U87-EGFRvIII glioma cells co-cultured with hMSC. However, the down-regulation of pAkt was most apparent in glioma cells co-cultured with hMSC-scFvEGFRvIII cells. No visible effect of co-culture of hMSCs on pAkt expression was observed in U87wt cells. These data are consistent with the growth characteristics of U87wt and U87-EGFRvIII cells in the presence of control and scFvEGFRvIII modified hMSCs. Recently, Huang and co-authors found that the EGFRvIII receptor cross-activates c-Met signaling pathway [37]. In separate set of experiments, we evaluated the activation of c-Met downstream signaling molecule STAT3 in U87-EGFRvIII cells co-cultured with control or scFvEGFRvIII modified hMSCs. We found that activation of STAT3 is suppressed in U87-EGFRvIII glioma cells co-cultured with hMSC-scFvEGFRvIII, but not control hMSCs or U87-EGFRvIII alone (Fig. S2A). It is important to note that STAT3 also controls cell growth and apoptosis. Therefore, down-regulation of pSTAT3 in these cells might be also partially responsible for this decrease in the growth of U87-EGFRvIII in presence of modified hMSCs. Interestingly, no significant change in the expression of EGFRvIII was detected in U87-EGFRvIII cells co-cultured with hMSC-scFvEGFRvIII (Fig. S2B). It is possible that decreased activation of downstream molecules Akt and STAT3 is the result of decreased autophosphorylation of the receptor itself.

### Effect of scFvEGFRvIII modification on the retention of hMSC in the tumor

**Figure 2:**
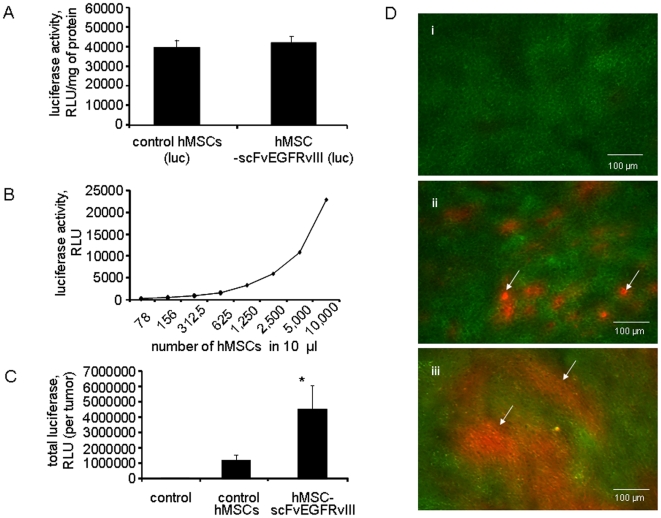
hMSC-scFvEGFRvIII presence in U87-EGFRvIII flank xenografts in athymic mice. A. hMSCs-scFv and control hMSCs were nucleofected with pGL4.14 plasmid encoding luciferase gene and stable population was selected with hygromycin. Control and scFvEGFRvIII expressing hMSC cells show equal expression of luciferase. Data presented as mean ± SD, n = 3. B. Titration of hMSC expressing luciferase. Sensitivity of assay. C. Presence of hMSC-vector and hMSCscFvEGFRvIII in the s.c. flank U87-EGFRvIII xenograft at day 25 either alone or after co-injection of control hMSC- vector or hMSC-scFvEGFRvIII cells (n = 8 in each group). Luciferase assay. Data presented as mean ± SD, * p<0.05. D. Microphotograph of 10 µm tissue sections from s.c. xenograft of (i) GFP expressing U87-EGFRvIII cells alone and either (ii) with control hMSC-vector or (iii) hMSC-scFvEGFRvIII cells at day 21. Arrows indicate the presence of hMSC expressing RFP.

In order to quantify the number of hMSCs within the tumor, hMSCs were nucleofected with a plasmid encoding firefly luciferase and selected with hygromycin to obtain the population of cells stably incorporating the luciferase gene. *In vitro*, luciferase assay demonstrated that control and scFvEGFRvIII modified hMSCs expressed equal amounts of luciferase (Figure 2A). We performed titration of hMSCs expressing luciferase to estimate the sensitivity of this assay and to determine the feasibility of its use for *in vivo* experiments. We found that hMSCs counts as low as 8,000 could be detected in the tumor homogenized in 1 ml of the buffer (Figure 2B).

We then injected 2×10^6^ hMSCs or hMSC-scFvEGFRvIII alone, into the right flank of athymic mice and confirmed that hMSCs themselves did not form tumors three weeks after injection (data not shown). Next, we investigated the influence of scFvEGFRvIII modification of hMSC on their retention in EGFRvIII expressing tumors *in vivo*. For this, U87-EGFRvIII glioma cells were injected alone, or with control hMSCs or hMSC-scFvEGFRvIII. On day 24, the tumors were excised, weighed and homogenized in a lysis buffer. The luciferase activity was measured in 10 µl of cleared supernatant. The total luciferase activity in the tumor sample (e.g. number of the stem cells present in the whole tumor) was recalculated by multiplying luciferase activity in 10 µl to the total volume of homogenate. Compared to flank tumors injected with control hMSCs, tumors injected with scFvEGFRvIII modified hMSCs displayed significantly increased luciferase activity (3.8 times more) (Figure 2C). No luciferase activity was detected in the control tumors injected with PBS. Assuming that luciferase activity in hMSC *in vitro* corresponds to that *in vivo*, it appears that flank tumors co-injected with 2×10^6^ hMSC-scFvEGFRvIII retain approximately the same number of the cells on day 24, whereas the number of control hMSCs is significantly decreased. It is unclear at this point if scFvEGFRvIII interaction with EGFRvIII on the surface of U87 cells promotes hMSCs survival and/or proliferation within the tumor. This will be the subject of further investigations.

**Figure 3:**
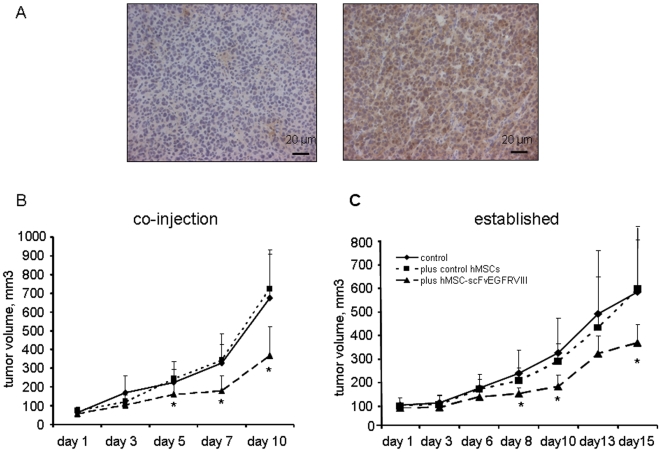
The effect of hMSC-scFvEGFRvIII on the growth of U87-EGFRvIII flank xenografts in athymic mice. A- Fixed paraffin embedded section from the flank U87-EGFRvIII xenografts were stained with anti-EGFRvIII mAb L8A4. Left panel is a section stained with isotype control mIgG. Right panel shows the expression of EGFRvIII in the tissues which was revealed with secondary antibodies conjugated with HRP. B- U87-EGFRvIII glioma cells alone or with equal amount of either control hMSC-vector or hMSC-scFvEGFRvIII (n = 8 in each group) were injected s.c. in the right flank and grew until became measurable for 14 days. The volume measurements were taken every other day for next 10 days. Data presented as mean ± SD, * p<0.05. C- U87-EGFRvIII glioma cells were injected s.c. in the right flank. After one week, PBS, control hMSCs or hMSC-scFvEGFRvIII (n = 6 in each group) (1×10^6^ cells in 100 µl of PBS) were injected in the middle of the tumor and grown for another week, after which the tumor volume measurements were taken every other day for the next 14 days. Data presented as mean ± SD, * p<0.05.

In order to visualize our quantitative findings, in a separate experiment, U87-EGFRvIII glioma cells expressing GFP and hMSCs expressing RFP were injected in to the right flank. After 3 weeks, the flank tumors were excised, flash frozen and freshly prepared 10 µm tissue sections were analyzed using fluorescent microscopy. Figure 2D(i) shows the GFP expressing glioma tumor cells in the presence of control hMSC expressing RFP (Figure 2D (ii)) or hMSC-EGFRvIII expressing RFP (Figure 3D (iii)) within the tumor bed. Representative picture out of 18 images captured from each block is shown. In accordance with our quantitative luciferase data, we observed a significantly higher presence of hMSC-scFvEGFRvIII (red fluorescence) in the tumor compared to control hMSCs. hMSCs were evenly distributed through the tumor and had an appearance of cellular islands rather than single hMSCs.

### Effect of scFvEGFRvIII modification of hMSCs on the tumor growth in *in vivo* models

In order to confirm our *in vitro* finding of delayed growth of U87-EGFRvIII glioma cells co-cultured with hMSC-scFvEGFRvIII, we performed several flank experiments.

In the first set of experiments, we studied the growth of s.c. flank xenografts after co-injection of U87-EGFRvIII glioma cells with hMSCs in athymic mice. Figure 3A shows staining of paraffin embedded tumor sections either with isotype control (left panel) or anti-EGFRvIII antibody (right panel), and confirms that flank tumors maintained expression of U87EGFRvIII. Two weeks following injection, tumors became measurable and tumor volume was assessed every other day for the next 10 days. We observed a significant delay in U87-EGFRvIII flank tumor growth in the presence of hMSC-scFvEGFRvIII (n = 8, p<0.05) by day 5. This growth clearly was not appreciated in either of the controls groups (Figure 3B). This disparity became more apparent with the progression of tumor growth. Tumor volume in the hMSC-scFvEGFRvIII group was approximately half the size of control tumors by the day 24 (p<0.05).

In a second set of experiments, we examined if hMSC-scFv could delay the growth of established s.c. flank U87-EGFRvIII tumors in athymic mice. After 1 week of growth, flank tumors reached a volume of 36±7 mm^3^ and received one of the following: (i) PBS (control); (ii) 1×10^6^ of control hMSCs; (iii) 1×10^6^ of hMSC-scFvEGFRvIII. One week following injection of hMSCs, the measurements of tumor volume were taken for the next 2 weeks and on day 28 the animals were sacrificed. Similar to the data in co-injection experiment described above, the U87-EGFRvIII flank tumors injected with hMSC-scFvEGFRvIII were 1.7 times smaller than tumors in the control (PBS) group (p = 0.03, n = 6) or tumors injected with control hMSCs (p = 0.05, n = 6) (326±79 vs 542±220 vs 555±267 mm^3^ respectively) at the end of experiment (Figure 3C).

### Effect of hMSC-scFvEGFRvIII on survival of animals with an intracranial model of U87-EGFRvIII glioma

**Figure 4:**
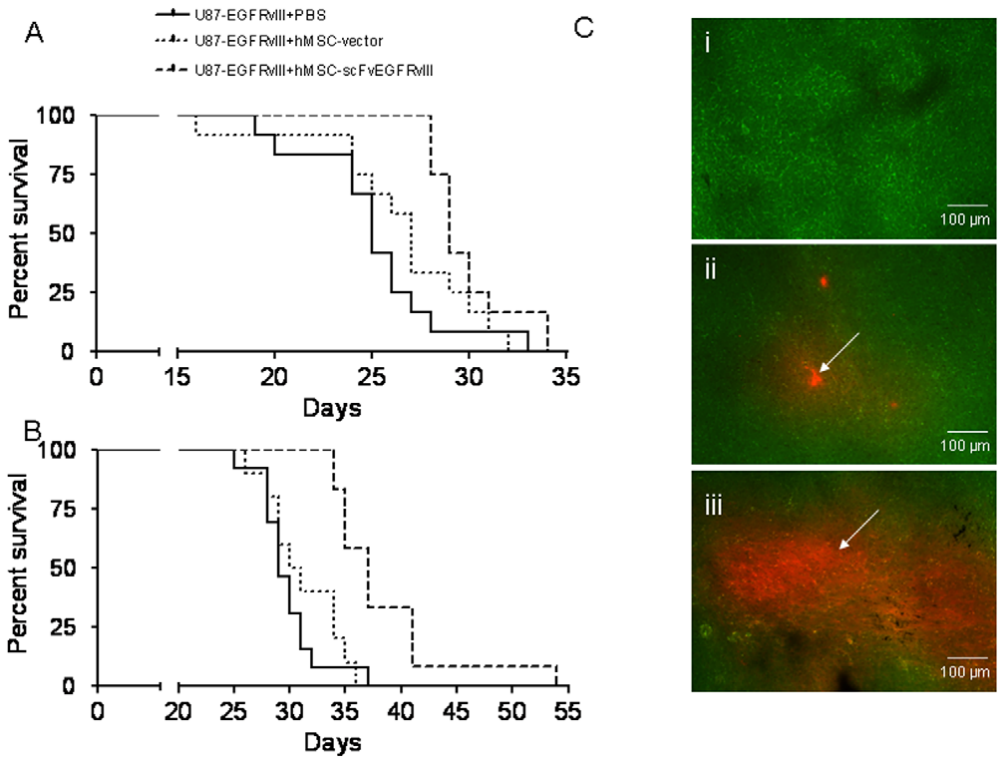
hMSC-scFvEGFRvIII extend the survival of U87-EGFRvIII glioma in an intracranial model. A. U87-EGFRvIII cells alone (1×10^5^) or with equal amount of control or scFvEGFRvIII expressing hMSCs (n = 12 per group) were inoculated in the right hemisphere of athymic mice. Survival of the animals was recorded until last animal died. B. U87-EGFRvIII cells alone (1×10^5^) (n = 13) or with equal amount of control (n = 10) or scFvEGFRvIII expressing hMSCs (n = 11) were inoculated in the right hemisphere of athymic mice. Animals received an additional 3×10^5^ of control or hMSC-scFvEGFRvIII intratumoral inoculation at 2 weeks and the survival of the animals was recorded. C. Microphotograph of 10 µm tissue sections from 4 week brain tumors (i) GFP expressing U87-EGFRvIII cells alone and either (ii) with control hMSC-vector or (iii) hMSC-scFvEGFRvIII cells. Arrows indicate the presence of hMSC expressing RFP.

In order to confirm our findings in s.c. flank xenografts model, we investigated the survival of animals injected intracranially with 1×10^5^ U87-EGFRvIII alone or co-injected with 1×10^5^ hMSCs. Figure 4A shows the survival curve. By day 27, only 17% and 42% of the animals survived in control group and in animals co-injected with control hMSCs, respectively. On the other hand, we observed 100% of survival in the animals injected with hMSC-scFvEGFRvIII. However, during week 5 (days 28 through 34), the survival of animals in hMSCs groups dropped drastically. Only one animal in control U87-EGFRvIII group lasted to the day 33. Pair-wise comparisons showed a significant difference in survival between the control (U87-EGFRvIII) group and those injected either with control hMSC (p<0.05, chi-square 5.32) or hMSC-scFvEGFRvIII (p<0.001, chi-square 19.03). The median survival of control animals or those injected with control hMSCs and hMSC-scFvEGFRvIII was 25, 27 and 29 days, respectively.

Keeping in mind that the initial number of injected hMSC-scFvEGFRvIII may not be sufficient to control the growth of exponentially growing tumor cells, we implanted an additional 3×10^5^ hMSCs into the brain tumors two weeks after the initial injection of 1×10^5^ U87-EGFRvIII and 1×10^5^ hMSCs and evaluated the survival of these animals (Figure 4B). The survival of the animals injected with hMSC-scFvEGFRvIII was increased in comparison to controls (p = 0.002, chi-square 121.24). The median survival of animals in control group or injected with control hMSCs and hMSC-scFvEGFRvIII was 29, 30.5 and 37 days, respectively. It is notable that the median survival in the group which received additional injection of hMSC-scFvEGFRvIII was extended over the group of animals without an additional injection, suggesting that hMSC expressing scFvEGFRvIII exert therapeutic efficacy on established intracranial tumors *in vivo*.

In order to evaluate the distribution of hMSCs within a brain tumor, tissues were flash frozen and cut into 10 µm sections for analysis. We observed significantly more robust presence of hMSC-scFvEGFRvIII in the U87-EGFRvIII expressing brain tumor than control hMSCs (Figure 4C). These data corresponded to our findings in the flank xenografts.

**Figure 5:**
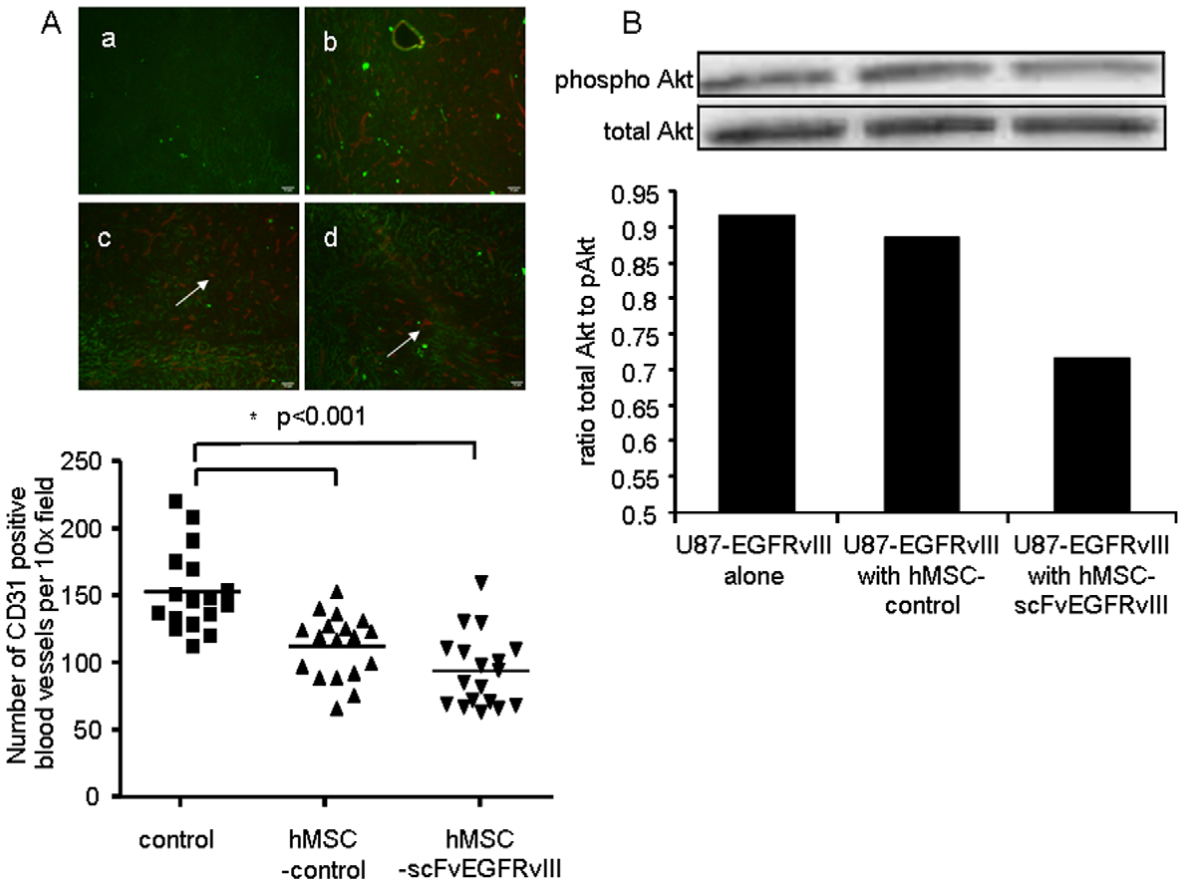
Brain tumor vascularization in the presence of hMSC-scFvEGFRvIII and pAkt expression. **A.** Tissue section (10 µm) from flash frozen brains carrying tumors were stained for mouse CD31, (a) negative control, U87-EGFRvIII GFP expressing tumor; (b) CD31 positive blood vessels in U87-EGFRvIII GFP expressing tumor; (c) CD31 positive blood vessels in U87-EGFRvIII GFP expressing tumor co-injected with control hMSCs; (d) CD31 positive blood vessels in U87-EGFRvIII GFP expressing tumor co-injected with hMSC-scFvEGFRvIII. Arrows show CD31 positive blood vessels. The number of CD31 positive blood vessels was calculated in each field (n = 18) (magnification 10×) and data summarized in the graph. * *p*<0.001. B. Expression of pAkt in glioma cells sorted from 4 week old tumors established with U87-EGFRvIII alone or with control or scFvEGFRvIII expressing hMSCs. Representative Western Blot from two independent experiments is shown.

Since MSCs are known to secrete pro-angiogenic factors, we evaluated if the significant retention of hMSCs expressing scFvEGFRvIII in the tumor tissue promoted an increase in tumor vascularization. Figure 5A shows there was no increase in the number of the CD31 positive blood vessels in tumors with hMSC-scFvEGFRvIII. In fact, we saw a lower number of CD31 positive blood vessels in both groups which received control hMSCs and hMSC-scFvEGFRvIII in comparison with a control group (73% and 61 % of control respectively, *p*<0.001). It also worth mentioning that we observed more prominent vascularization in brain tumors compared to flank xenografts (data not shown)

Finally, we investigated if the inhibition of pAkt which we observed *in vitro* is also sustained *in vivo*. Four week brain tumors were excised and strained through the 70 µm meshworks. GFP positive U87-EGFRvIII cells were sorted and analyzed by gel-electrophoresis and Western Blot for pAkt and total Akt expression. We found no difference in total Akt between the groups. However, pAkt was down-regulated by 28% in glioma cells co-injected with hMSC-scFvEGFRvIII. No difference in pAkt expression in glioma cells was observed between control and control hMSCs group (Figure 5B). Thus, these data are in agreement with the delay of the growth observed in U87-EGFRvIII tumors in the presence of hMSC-scFvEGFRvIII and an increase in survival of these animals. It is also consistent with our microscopy findings which show that hMSC expressing scFvEGFRvIII present in tumor are in touch with only a fraction of glioma cells within the tumor.

## Discussion

Our previous study demonstrated that hMSCs can be genetically engineered to express scFvEGFRvIII on the cell surface and show improved binding with EGFRvIII expressing U87 glioma cells [36]. In this study, we investigated if genetically modified hMSCs expressing scFvEGFRvIII on their surface show an increased retention in EGFRvIII expressing experimental glial tumors and whether these cells can modify tumor growth through antibody single-chain fragment-antigen interaction.

Here, we demonstrated that scFvEGFRvIII expressing hMSCs were retained in U87-EGFRvIII xenografts approximately four times better than control hMSCs. Our quantitative data are in a strong agreement with microscopy analysis of tissue sections from both flank xenografts and intracranial brain tumors, showing significant presence of hMSC-scFvEGFRvIII within the tumor mass in comparison with control hMSCs. Thus, genetic modification of hMSCs with small antibody fragments to tumor specific antigens opens the window for improvements in stem cell based therapies of cancers overall.

In this study, we investigated if scFvEGFRvIII expressing hMSCs could also influence the growth of U87 glioma cells expressing EGFRvIII *in vitro*. Interestingly, we found that both control hMSCs and hMSC-scFvEGFRvIII did not influence the growth of U87wt cells. However, hMSC-scFvEGFRvIII delayed the growth of U87-EGFRvIII glioma cells about 1.6 times more in comparison with control hMSCs. We attribute this effect to the interaction of scFvEGFRvIII expressed on the surface of hMSCs with EGFRvIII expressed by the glioma cells. In this respect, Sampson and co-authors demonstrated that a single intratumoral injection of murine antibody Y10, which recognizes EGFRvIII, significantly increased the median survival of animals with brain B16 melanoma tumors by 286% [21]. The mAb 806, which specifically recognizes U87 expressing EGFRvIII, was able to inhibit the growth of xenografts in a dose dependent manner, but not U87wt flank xenograft tumors [38]. Moreover, the significant inhibition of EGFRvIII expressing brain tumors in the intracranial model was observed after systemic injection of mAb806 [22].

We found that the delay in the growth of U87-EGFRvIII cells in co-culture with hMSC-scFvEGFRvIII is mediated in part through down-regulation of pAkt in U87 cells. We also observed the down-regulation of pAkt in brain tumor samples. The down-regulation of pAkt *in vitro* was greater than *in vivo*. This is consistent with the fact that *in vitro*, most U87-EGFRvIII glioma cells are in intimate contact with hMSC-scFvEGFRvIII. *In vivo*, however, we observed a patchy pattern of distribution of hMSC-scFvEGFRvIII in the tumor. This suggests that only a fraction of U87-EGFRvIII glioma cells were in contact with the stem cells. Phospho-Akt executes its role in tumorgenesis through inhibition of apoptosis and mediation of proliferation. It has been shown that GBMs expressing EGFRvIII demonstrate increased growth and invasiveness mostly through the PI3K/pAkt [37]. Huang and co-authors also found that c-Met signaling pathway was cross-activated in EGFRvIII over-expressing cells [37]. In our studies, we also observed a decrease in activation STAT3 in U87-EGFRvIII cells co-cultured with hMSCs-EGFRvIII, suggesting that multiple pathways might be involved in the regulation of growth in these cells. No significant changes in the expression of the EGFRvIII receptor were observed in our studies. It is possible that decreased activation of downstream molecules Akt and STAT3 is caused by decreased autophosphorylation of the receptor itself. Mishima and co-authors investigated the effect of a monoclonal anti-EGFRvIII antibody (mAb 806*) in vivo*. They demonstrated that treatment with mAb 806 resulted in a significant decrease in autophosphorylation of EGFRvIII and downstream molecule Bcl-X_L_, whereas the receptor level was only slightly decreased. The authors concluded that this particular antibody affected the intrinsic signaling function of the EGFRvIII receptor [22]. Interestingly, in our *in vitro* study, pAkt was also down-regulated in U87-EGFRvIII by control hMSCs although to a lesser extent than by hMSC-scFvEGFRvIII. This was not observed *in vivo*. It has been shown that hMSCs were able to down-regulate pAkt in Kaposi's sarcoma cells when co-cultured, as well as in primary endothelial cells and neonatal cardiac myocytes, and this effect required direct cell-cell contact [39].

To support our findings *in vitro*, we evaluated the growth of U87-EGFRvIII s.c. flank xenografts alone or in the presence of control or scFvEGFRvIII expressing hMSCs. Similar to our *in vitro* data, flank xenografts grew at significantly slower rate in the presence of hMSCs expressing scFv EGFRvIII than control tumors. This delay in U87-EGFRvIII flank xenografts growth was similar when glioma cells and hMSCs were co-injected or hMSCs injected into established tumor. It is worth noting that the effect of hMSC-scFvEGFRvIII on established EGFRvIII tumor was dependent on the initial tumor size. When hMSCs-scFv were injected in larger tumors, they did not influence xenograft growth. This could be due to the fact that the proportion of injected hMSCs to the tumor mass is relatively low. With this in mind, repeated inoculations of hMSCs in the tumors and possibly at different locations might be necessary for efficient therapy of large established tumors. Other authors have made the observation that the therapeutic effect of MSCs is dependent on the proportion of hMSCs to the tumor cells [34]. A recent study by Kim and co-authors also suggested that repeated administration of MSC-stTRAIL in established glioma tumor with a control of cell number, interval between injection and expression of the therapeutic protein will be necessary for clinical application [35]. We observed no effect of control hMSCs on the growth of flank or brain tumors. These data are in an agreement with recent study demonstrating the absence of the growth-promoting effect of MSCs on glial brain tumors [31], which was shown in other types of tumors [40], [41]. Of note, while there is clear heterogeneity in EGFRvIII expression in brain tumors, the enhanced benefit of targeting modified hMSC should result in a bystander effect to cells which do not express EGFRvIII. The prolonged association of these MSC with the tumor may translate in the prolonged release of secreted therapeutics in the tumor environment.

In our study the survival of animals with intracranial of U87-EGFRvIII glioma co-injected with hMSC-scFvEGFRvIII was significantly improved compared to the control groups. However, the survival was prolonged only by one week. An additional injection of hMSC-scFvEGFRvIII in the already growing tumor further prolonged the median survival of the animals. These data suggest that tumor-eradicating therapy will have to take advantage of the substantial presence of hMSC-scFvEGFRvIII within the tumor in comparison with unmodified hMSCs. Interestingly, we found a lower density of CD31 positive blood vessels in the brain tumors from the animals that received control hMSCs and hMSC-scFvEGFRvIII. This was also consistent with the increase in animal survival in these groups in comparison with controls and can be explained by the delay of tumor growth and therefore lower density of the CD31 positive blood vessels at given time points. The literature on the effect of MSCs on neovascularization of the tumors, however, is controversial. It has been reported that MSCs are the source of soluble factors promoting angiogenesis [42], [43]. However, it also has been reported that MSCs are able to decrease angiogenesis in melanoma tumor xenografts model [44], but not affect the angiogenesis in rat malignant glioma [45]. The effect of MSCs on angiogenesis therefore likely depends on a particular tumor type.

In conclusion, we demonstrate for the first time that hMSCs modified to express scFv to EGFRvIII on their surface displayed prolonged retention in EGFRvIII expressing glioma. This was confirmed *in vivo* in flank and intracranial xenografts models. Moreover, we found that hMSC-scFvEGFRvIII posses therapeutic effect by delaying growth of tumors and prolonging the survival of the animals with intracranial brain tumors. However, this “bonus” property of hMSC-scFvEGFRvIII is not sufficient to use these cells as monotherapy. Taking in consideration the increased presence of hMSCs expressing scFvEGFRvIII within the tumor, the modification of these stem cells with a therapeutic gene might significantly boost their therapeutic potential. The use of genetically modified hMSC with scFv to EGFRvIII and other tumor specific antigens might eliminate the need of repetitive inoculation of hMSC to achieve the prolonged and desired therapeutic effect. Such an approach offers an attractive prospect for gene therapy applications and should be further explored in preclinical tumor models.

## Materials and Methods

### Ethics Statement

All studies were reviewed and approved by the Institutional Animal Care Committee at the University of Chicago.

### Reagents

MEM and MEM alpha media, antibiotic-antimycotic were purchased from Invitrogen (Carlsbad, CA). The premium select fetal bovine serum (FBS) was obtained from Atlanta Biologicals (Lawrenceville, GA). Trypsin-EDTA was purchased from Mediatech Inc. (Manassas, VA). The geneticin (G418), 5-bromo-2′-deoxyuridine (BrdU) and anti-actin antibodies were purchased from Sigma (St. Louis, MO). The fluorescent tracer carboxyfluorescein diacetate, succinimidyl ester (CFDA SE) was purchased from Molecular Probes (Eugene, OR). BrdU flow kit was obtained from BD Biosciences (San Diego, CA). M-Per mammalian protein extraction buffer, Halt protease inhibitors cocktail and phosphatase inhibitor are from Thermo Scientific (Rockford, IL). The Tris-HCl gel and PVDF membrane, ImmunStar WesternC were purchased from Bio-Rad, (Hercules, CA). Antibodies to total and pAkt were purchased from Cell Signaling Technology (Danvers, MA). Antibodies to phospho STAT3 and total STAT3 were obtained from Santa Cruz Biotechnology (Santa Cruz, CA). Secondary anti-mouse and anti-rabbit antibodies conjugated peroxidase were purchased from Chemicon Intl. (Temicula, CA). The rat anti-mouse CD31 antibodies were obtained from Santa Cruz Biotechnology (Santa Cruz, CA) and secondary anti-rat antibodies conjugated with DyLight 649 were obtained from Jackson ImmunoResearch Laboratories (West Grove, PA). The monoclonal antibody (mAb) L8A4 is generous gift of Dr. Bigner (Duke University Medical Center, Durham, NC). The plasmid pGL4.14 encoding luciferase was purchased from Promega (Madison, WI).

### Cell Culture

hMSC were grown in MEM alpha media supplemented with antibiotic-antimycotic and 10% premium select FBS. Media was replaced every 48 hours. Cells were re-plated using 0.25% trypsin-EDTA after they reached 80% of confluency. The U87 glioma cells expressing EGFRvIII were grown in MEM media supplemented with 10% heat inactivated FBS and 200 µg/ml of G418.

### Genetic Modification of hMSC

hMSCs were modified to express scFv to EGFRvIII as previously described [36]. Briefly, hMSCs were nucleofected either with empty vector (control hMSCs) or with a plasmid encoding scFv EGFRvIII-PDGFR transmembrane domain fusion using AMAXA technology. The stable population of cells expressing scFvEGFRvIII was selected with G418 and further enriched using cell sorting. The control hMSCs and hMSC-scFvEGFRvIII were infected with retrovirus encoding red fluorescent protein (RFP) and selected with puromycin. For luciferase expression, hMSCs were nucleofected with pGL4.14 plasmid encoding luciferase gene and stable population of cells that incorporated the luciferase gene was selected with hygromycin.

### Proliferation assay

GFP negative U87wt and U87-EGFRvIII cells were labeled with 2.5 µM SFDA SE fluorescent tracer and immediately seeded on 6 well plates at density of 1×10^5^ cells/well. The control hMSC or hMSC-scFvEGFRvIII were added to the U87 cells with following ratios: 1∶0; 1∶0.1; 1∶1 and 1∶10. Culture media was changed every 48 hours. After 5 days, the cells were collected using trypsin-EDTA and SFDA SE labeled U87 cells were analyzed by flow cytometry. Mean fluorescence intensity (MFI) was estimated for each group of co-cultured cells. In order to demonstrate the specificity of the effect hMSC-scFvEGFRvIII on the growth of U87-EGFRvIII cells, data were presented as ratio of MFI for U87 cells co-cultured with hMSC-scFvEGFRvIII to that with control hMSCs.

### BrdU Assay

Stable populations of control and scFvEGFRvIII expressing hMSCs were pulsed with 10 µM BrdU for 2 hours. Cells were treated with only BrdU solvent as a control for background binding of anti-BrdU-FITC mAb with cells. Cells were stained for incorporated BrdU according to manufacturer's recommendation. Briefly, cells were collected and fixed with fixation/perm solution. Washed cells were treated with DNase for 1 hour at 37°C and after washing were subsequently stained with anti-BrdU-FITC antibodies. Percentage of BrdU positive cells was determined by flow cytometry.

### Western Blotting

U87wt or U87-EGFRvIII cells expressing green fluorescent protein (GFP) were co-cultured either with control hMSCs or hMSC-scFvEGFRvIII at the ratio 1∶1 for 48 hours. Co-cultured cells were dissociated using trypsin-EDTA and sorted to collect only GFP positive U87 cells. To analyze the U87-EGFRvIII cells from the brain tumors, the tumors were strained through 70 µm meshworks and sorted to collect GFP positive U87 cells. The cell pellets were lysed using the M-Per mammalian protein extraction buffer containing Halt protease inhibitors cocktail and phosphatase inhibitor. Samples were applied to 10% Tris-HCl gel at 40 µg/lane and resolved under reducing conditions. After the transfer of proteins to PVDF membrane and blocking with 2% non-fat dry milk, the membrane was stained subsequently with anti-pAKT, total Akt, anti-pSTAT3, total STAT3, anti-EGFRvIII and anti-beta actin antibodies, followed by appropriate secondary antibody conjugated with peroxidase. ImmunStar WesternC was used to develop the reaction. Images were captured using the Bio-Rad's ChemiDoc Imaging System (Hercules, CA).

### Immunocytochemistry

Flash frozen flank and brain tumor tissues were cut to a thickness of 10 µm. Tissue sections were fixed with −20°C methanol and stained for mouse CD31 using rat monoclonal antibodies at concentration 5 µg/ml. The bound antibodies were revealed by goat anti-rat DyLight649 secondary antibodies at concentration 10 µg/ml. After repeated washing to remove unbound antibodies with PBS containing 0.05% Tween 20, cover slips were rinsed once in water and mounted in fluoromount-G (Southern Biotech, Birmingham, Alabama) containing 1 µg/ml DAPI. Positive FITC staining was analyzed using Olympus IX70 inverted microscope and MetaMorph software.

For EGFRvIII staining, xenograft tissue was fixed with 4% paraformaldehyde. Paraffin-embedded tissue was cut to 6 µm and antigen retrieval was performed using 0.01 M citrate buffer at pH 6.0 for 30 minutes in the oven. Tissue sections were stained with 10 µg/ml of L8A4 mAb and revealed with secondary anti-mouse antibody conjugated with HRP.

### Animal Studies

All animals were maintained and cared for in accordance with the Institutional Animal Care and Use Committee protocol and regulations. The animals used in the experiments were 6–8 weeks old male athymic nu/nu mice from Harlan Laboratories (Indianapolis, IN). All animals were housed in a pathogen free facility at The University of Chicago. Mice were anaesthetized with an intraperitoneal injection of ketamine hydrochloride (25 mg/ml)/xylazine (2.5 mg/ml) cocktail.

For s.c. xenografts, mice were injected in the right flank with 2×10^6^ of U87-EGFRvIII cells in 100 µl of PBS or in a mixture with equal amount of control hMSCs or hMSCscFv. In a separate set of experiments, PBS, control hMSCs, or hMSC-scFvEGFRvIII were injected into the tumor in growing U87-EGFRvIII flank xenografts. When tumors became measurable, tumor dimensions were estimated using slide caliber and the volume was calculated according the previously reported formula (L x W x H)/2 [46].

To establish intracranial tumors, a midline cranial incision was made and a right-sided burr hole was placed 2 mm lateral to the sagittal sinus and approximately 2 mm superior to the lambda. Animals were positioned in stereotactic frame and a Hamilton needle was then inserted into the burr hole and advanced 3 mm. Intracranial penetration was followed by injection of 1×10^5^ cells in 2.5 µl of sterile PBS. In one set of experiments, U87-EGFRvIII cells were injected alone or in mixture with 1×10^5^ MSC-vector or hMSC-scFvEGFRvIII. In another set of experiments, animals received additional injection of 2.5 µl PBS or 3×10^5^ of hMSCs in the same volume (control hMSCs or hMSC-scFvEGFRvIII). All mice were followed to assess survival and upon death, the brains were harvested for microscopic analysis.

### Statistical analysis

The differences between groups were evaluated by calculating Student's *t*-value or one way ANOVA with post-hoc comparison Tukey's test. For the *in vivo* survival data, a Kaplan-Meier survival analysis was used and statistical analysis was performed using a Log rank test. *P*<0.05 was considered statistically significant.

## Supporting Information

Figure S1 Comparison of control hMSCs and scFvEGFRvIII modified MSCs with respect to the rate of proliferation in vitro. Control and scFvEGFRvIII modified MSCs were labeled with BrdU for 2 hours at 370C. After the fixation/permeabilization and treatment with DNase, control and BrdU treated cells were stained anti-BrdU-FITC antibody and analyzed by flow cytometry. Data is presented as percentage of BrdU positive cells (mean ± SD). Summary of two independent experiments is shown.
(0.12 MB TIF)

Figure S2 A. Phosphorylation of STAT3 in U87-EGFRvIII cells after co-culture with hMSC-scFvEGFRvIII. U87-EGFRvIII cells expressing GFP and hMSCs were co-cultured at equal ratio for 48 hours. The U87-EGFRvIII cells were sorted out based on their GFP expression and cell pellets were processed for gel electrophoresis and Western Blot analysis. The phosphorylated STAT3 (phoso STAT3), total STAT3 and beta actin in cell lysates were detected using primary antibodies and developed with secondary antibodies conjugated with HRP. Representative blots of two independent experiments are shown. B. Expression EGFRvIII in U87-EGFRvIII cells after co-culture with hMSC-scFvEGFRvIII. U87 cells expressing GFP and hMSCs were co-cultured at equal ratio for 48 hours. The U87wt and U87-EGFRvIII cells were sorted out based on their GFP expression and cell pellets were processed for gel electrophoresis and Western Blot analysis. The EGFRvIII and beta actin in cell lysates were detected using primary antibodies and developed with secondary antibodies conjugated with HRP. A representative blot of two independent experiments is shown.
(1.41 MB TIF)
