# Supplementary figures and images for: Mesenchymal Stem Cells Modified with a Single-Chain Antibody against EGFRvIII Successfully Inhibit the Growth of Human Xenograft Malignant Glioma
Source: PLoS One. 2010 Mar 18;5(3):e9750. doi: 10.1371/journal.pone.0009750 (PMC2841188; doi:10.1371/journal.pone.0009750)

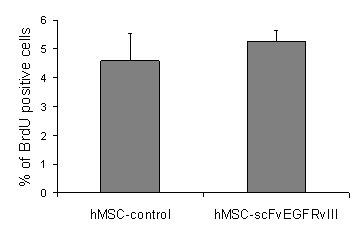

Supplement: Figure S1 — Comparison of control hMSCs and scFvEGFRvIII modified MSCs with respect to the rate of proliferation in vitro. Control and scFvEGFRvIII modified MSCs were labeled with BrdU for 2 hours at 370C. After the fixation/permeabilization and treatment with DNase, control and BrdU treated cells were stained anti-BrdU-FITC antibody and analyzed by flow cytometry. Data is presented as percentage of BrdU positive cells (mean ± SD). Summary of two independent experiments is shown. (0.12 MB TIF) [file pone.0009750.s001.tif]

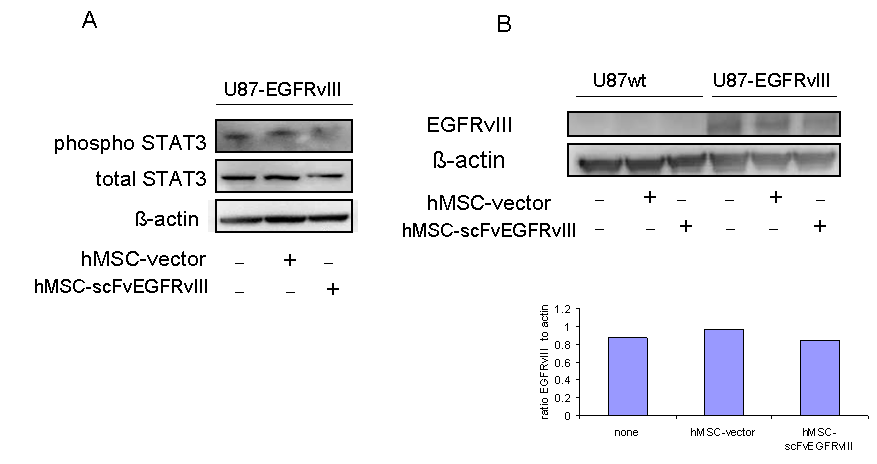

Supplement: Figure S2 — A. Phosphorylation of STAT3 in U87-EGFRvIII cells after co-culture with hMSC-scFvEGFRvIII. U87-EGFRvIII cells expressing GFP and hMSCs were co-cultured at equal ratio for 48 hours. The U87-EGFRvIII cells were sorted out based on their GFP expression and cell pellets were processed for gel electrophoresis and Western Blot analysis. The phosphorylated STAT3 (phoso STAT3), total STAT3 and beta actin in cell lysates were detected using primary antibodies and developed with secondary antibodies conjugated with HRP. Representative blots of two independent experiments are shown. B. Expression EGFRvIII in U87-EGFRvIII cells after co-culture with hMSC-scFvEGFRvIII. U87 cells expressing GFP and hMSCs were co-cultured at equal ratio for 48 hours. The U87wt and U87-EGFRvIII cells were sorted out based on their GFP expression and cell pellets were processed for gel electrophoresis and Western Blot analysis. The EGFRvIII and beta actin in cell lysates were detected using primary antibodies and developed with secondary antibodies conjugated with HRP. A representative blot of two independent experiments is shown. (1.41 MB TIF) [file pone.0009750.s002.tif]
